# Depression mediates the association between health literacy and health-related quality of life after myocardial infarction

**DOI:** 10.3389/fpsyt.2024.1341392

**Published:** 2024-02-14

**Authors:** Inge Kirchberger, Simone Fischer, Philip Raake, Jakob Linseisen, Christine Meisinger, Timo Schmitz

**Affiliations:** ^1^ Epidemiology, Faculty of Medicine, University of Augsburg, Augsburg, Germany; ^2^ Department of Cardiology, Respiratory Medicine and Intensive Care, University Hospital Augsburg, Augsburg, Germany; ^3^ Institute for Medical Information Processing, Biometry and Epidemiology (IBE), Ludwig-Maximilians Universität Munich, Munich, Germany

**Keywords:** health literacy, health-related quality of life, myocardial infarction, depression, education

## Abstract

**Introduction:**

So far, health literacy (HL) and its related factors in patients with acute myocardial infarction received little attention. Thus, the objective of this study was to investigate the associations between the different dimensions of HL and disease-specific health-related quality of life (HRQOL), and factors that may affect these relations in patients after acute myocardial infarction (AMI).

**Methods:**

All survivors of AMI between June 2020 and September 2021, from the Myocardial Infarction Registry Augsburg (n=882) received a postal questionnaire on HL [Health Literacy Questionnaire (HLQ)], HRQOL (MacNew Heart Disease HRQOL questionnaire) and depression (Patient Health Questionnaire). From the 592 respondents, 546 could be included in the analysis. Multivariable linear regression models were performed to investigate the associations between the nine subscales of the HLQ and the total score and three subscales of the MacNew questionnaire. A mediation analysis was performed to estimate direct and indirect effects of HL on HRQOL taking into account the mediating effect of depression.

**Results:**

In the sample of 546 patients (72.5% male, mean age 68.5 ± 12.2 years), patients with poor education showed significantly lower HLQ scores. Significant associations between the subscales of the HLQ and the MacNew were found, which remained significant after adjustment for sociodemographic variables with few exceptions. More than 50% of the association between HL and HRQOL was mediated by depression in seven HLQ subscales and a complete mediating effect was found for the HLQ subscales ‘Actively managing my health’ and ‘Appraisal of health information’.

**Discussion:**

Depression mediates the associations between HL and disease-specific HRQOL in patients with myocardial infarction.

## 1 Introduction

The concept of health literacy is increasingly recognized as a key determinant of health, both in healthy people and those with diverse diseases (1). Health literacy generally refers to competencies of accessing, understanding, appraising and applying health-related information within health care, disease prevention and health promotion settings (1, 2). Overall, diverse studies have found a consistent adverse relationship between low health literacy and participation in health promotion and prevention activities, health-promoting behaviors, and active management of chronic diseases (1).

In terms of cardiovascular diseases (CVD), studies found proportions that 32.8% of patients with CVD exhibit low health literacy (3), 30.5% in coronary artery disease (4) and 39% in heart failure (5). Patients with low health literacy were more likely to be older, unemployed, male, from a non-white ethnic group, to have more comorbidities, and a poorer education and socioeconomic position (4). Systematic reviews and meta-analyses showed that low health literacy was significantly associated with impaired health-related quality of life (HRQOL) (3, 4), higher anxiety and lower social support (4), less adherence to medical treatment (6), increased hospital readmission (3, 4), and increased mortality (3). However, there is little knowledge about factors that affect the association between health literacy and HRQOL. For instance, depression was shown to be strongly associated with both HRQOL (7) and health literacy (8), and was reported to mediate the relationship between health literacy and medication- or diet adherence, self-care and HRQOL (9–11). However, such mediating effects have not yet been investigated in persons with acute myocardial infarction and HRQOL as outcome.

A systematic review of the instruments used in studies investigating health literacy in CVD showed that most studies had a limited measurement approach and exclusively assessed reading ability and comprehension skills, and numeracy (12, 13). However, other questionnaires which better reflect the broader concept of health literacy are already available. For instance, the Health Literacy Questionnaire (HLQ) covers nine different domains of health literacy and therefore provides a comprehensive description of health literacy (14). So far there are only a few studies which applied a selection of two (15) or four domains (16, 17) of the HLQ, and only two studies used all nine domains (18, 19).

In addition, studies which investigated the association between health literacy and HRQOL used a generic assessment of HRQOL instead of a questionnaire specific to CVD and mostly had samples consisting of diverse cardiovascular disorders (3). Studies on patients with acute myocardial infarction which provided a comprehensive assessment of health literacy and disease-specific HRQOL are missing so far. However, a detailed knowledge of the associations between health literacy and HRQOL and factors which may affect these associations is crucial for the development of interventions in order to promote health literacy and improve outcomes after acute myocardial infarction (20).

Thus, the objective of the present study was to investigate the associations between the different dimensions of health literacy and disease-specific HRQOL and factors that may affect these relations in patients after acute myocardial infarction.

## 2 Methods

### 2.1 Design and study population

The present study used data from a postal follow-up survey on participants of the Augsburg Myocardial Infarction Registry, which was established as a part of the MONICA-project (Monitoring Trends and Determinants in Cardiovascular disease) in 1984. The study area covers the city of Augsburg, Germany, and the two adjacent counties (rural districts of Augsburg and Aichach-Friedberg), including a total of approximately 680,000 inhabitants. The registry continuously registers all cases of coronary death and non-fatal acute myocardial infarction of the study population older than 24 years. Methods of case finding, diagnostic classification of acute myocardial infarction as well as data quality control were detailed elsewhere (21, 22).

The registry was approved by the ethics committee of the Bavarian Medical Association (Bayerische Landesärztekammer) and the study was performed in accordance with the Declaration of Helsinki. Written informed consent was obtained from all participants.

In November 2021, all survivors with incident or recurrent myocardial infarction admitted between June 1st, 2020, and September 15th, 2021 (n = 882) were sent a questionnaire via post and were asked to complete questions on SarsCoV-2 vaccination, COVID-19 disease, diabetes, use of complementary therapies, HRQOL, depression, fatigue, and health literacy. Patient who did not respond within four weeks (n = 504), received a postal reminder on December, 14^th^, 2021. A total of 592 (67.1%) patients returned the questionnaire, 33 patients had died, 16 were not able to answer the questions due to dementia, 8 declined participation, and 233 patients did not respond. From the 592 respondents, 46 were excluded [acute myocardial infarction diagnosis not confirmed (n = 8), acute myocardial infarction before 2020 (n = 3), empty questionnaire (n = 7), missing scores of health literacy or HRQOL (n = 28)], leaving 546 cases for the analysis.

### 2.2 Survey data

Health literacy was assessed using the ‘Health Literacy Questionnaire’ (HLQ). The HLQ consists of 44 items covering nine domains of health literacy (14), namely: ‘1. Feeling understood and supported by healthcare providers’ (4 items), ‘2. Having sufficient information to manage my health’ (4 items), ‘3. Actively managing my health’ (5 items), ‘4. Social support for health’ (5 items), ‘5. Appraisal of health information’ (5 items), ‘6. Ability to actively engage with healthcare providers’ (5 items), ‘7. Navigating the healthcare system’ (6 items), ‘8. Ability to find good health information’ (5 items), and ‘9. Understanding health information well enough to know what to do’ (5 items). HLQ scores range between 1 and 4 for the first 5 scales, and 1 and 5 for scales 6 to 9 with higher scores indicating better health literacy. The HLQ demonstrated good measurement properties in several settings and populations (14, 23, 24). For the German version, the psychometric quality of the instrument has been confirmed (25).

To assess HRQOL, the ‘McNew Heart Disease Health-related Quality of Life Instrument’(MacNew) was used (26). The McNew consists of 26 items assigned to three subscales, namely ‘Physical’, ‘Emotional’ and ‘Social’. In addition, a global score can be calculated from all items. The scores range between 1 and 7 with higher values indicating better HRQOL (27). The German version of the McNew was validated in patients with acute myocardial infarction and demonstrated satisfactory reliability, validity and sensitivity (28, 29).

Depressive symptoms were assessed with the depression module of the Patient Health Questionnaire (PHQ) (30–32). The PHQ-9 consists of 9 items with response options from 0 to 3 (never to nearly every day), resulting in a score ranging from 0 to 27. A score less than five can be interpreted as the absence of depressiveness. Values between five and ten constitute a mild degree of depressiveness. Values of ten and higher can be subdivided into moderate (ten to 14), moderately severe (15 to 19), and severe (20 to 27) depressiveness (30). The German version of the PHQ-9 showed good psychometric properties (33).

### 2.3 Demographic and clinical data

Demographic characteristics and health characteristics such as body mass index (BMI), acute myocardial infarction risk factors, acute myocardial infarction type, acute myocardial infarction treatment, and co-morbidities were routinely obtained by patient interview and extracted from medical records by study nurses during the hospital stay for the acute myocardial infarction.

### 2.4 Data analysis

Continuous variables were described as means ± standard deviations (SD) and categorical variables as absolute and relative frequencies. Subgroup differences in HLQ scores were tested using Mann-Whitney U-Test, Kruskal-Wallis test, t-Test or analysis of variance, if appropriate. Multivariable linear regression models were performed to investigate the associations between the different dimensions of health literacy (independent variables) and the HRQOL (total score, and emotional, social and physical subscale) as dependent variables. First, unadjusted regression models were calculated. Second, the models were adjusted for age, gender, school education, nationality and living alone. Finally, the PHQ score was included as additional covariable in the models.

**Figure 1:**
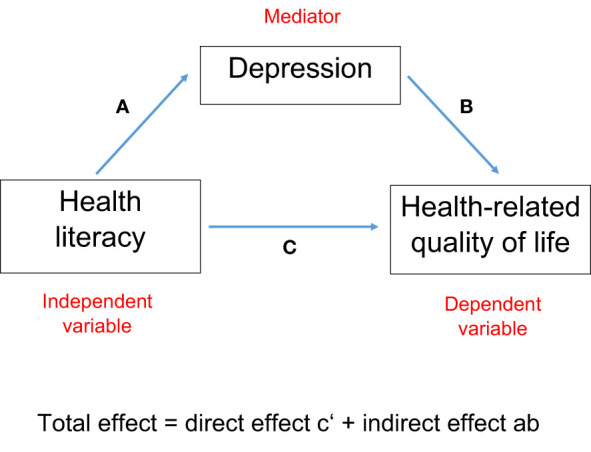
Model of the mediating effect of depression on the association between health literacy and health-related quality of life.

Since the results of the regression models indicated a mediating effect of the PHQ score, a mediation analysis was performed. The indirect effect of depression was calculated as the product of the ß regression estimates derived from linear regression of health literacy (independent variable) and depression (dependent variable) (path a), and depression (independent variable) and HRQOL (dependent variable) (path b) (see **Figure 1**). Path c’ refers to the direct association between health literacy and HRQOL estimated by fully adjusted regression models. The total effect is the sum of the direct and indirect effect (c’ + (a*b)).

The assumptions of multivariable linear regression were ensured. For the regression models, we report the linear regression coefficient ß, its 95% confidence interval and the corresponding p-value. A positive ß coefficient suggests that as the value of the independent variable increases, the mean of the dependent variable also increases. A negative ß coefficient indicates that as the independent variable increases, the dependent variable tends to decrease. The coefficient value shows how much the mean of the dependent variable changes given a one-unit shift in the independent variable while holding other variables in the model constant. We also report the 95% confidence interval of ß, which indicates the range of values within which we can be 95% certain that the true effect lies.

An alpha level of 0.05 was defined for the statistical tests. Although a large number of tests and regression models were conducted, we did not adjust for multiple testing due to the explorative character of these analyses. Statistical analyses were performed using SAS Version 9.4.

## 3 Results

### 3.1 Sample characteristics

The demographic and clinical characteristics of the participants are shown in **Table 1**. The mean age of the enrolled participants was 68.5 ± 12.2 years with 72.5% men and 27.5% women. Most of the participants had German nationality (92.9%) and no migration background (87.5%). School education of longer than 9 years was reported by 43.4% of the participants and only one third of the participants (31.1%) were still employed. Most of the participants were married (69.7%) and 22.2% were living alone. The most common comorbidity was hypertension (74.0%). About one half of the participants (50.5%) showed mild to severe depressiveness according to the PHQ-9.

**Table 1:** Sample characteristics.

	N total	Mean ± SD
Age (years)	546	68.5 ± 12.2
		n (%)
Male sex	546	396 (72.5)
German nationality	546	507 (92.9)
Migration background	537	67 (12.5)
School education > 9 years	539	234 (43.4)
Employed	543	169 (31.1)
Living alone	544	121 (22.2)
Married	544	379 (69.7)
Hypertension	546	404 (74.0)
Dyslipidemia	546	295 (54.0)
Diabetes	546	149 (27.3)
Obesity (>30 kg/m²)	546	143 (26.2)
Prior stroke	546	42 (7.7)
Prior infarction	479	73 (15.2)
**Type of infarction**	518	
STEMI		229 (44.2)
NSTEMI		241 (46.5)
Bundle branch block		48 (9.3)
**Smoking status**	544	
Smoker		127 (23.4)
Ex-Smoker		207 (38.1)
Never smoker		210 (38.6)
Any recanalisation therapy	546	506 (92.7)
Rehabilitation	537	320 (59.6)
**Depression** ^1^	545	
no		270 (49.5)
mild		177 (32.5)
moderate		69 (12.7)
severe		29 (5.3)
Health-related Quality of Life^2^		Mean ± SD
Physical	546	5.5 ± 1.2
Emotional	546	5.4 ± 1.1
Social	546	5.7 ± 1.1
Total	546	5.5 ± 1.1

^1^Patient Health Questionnaire¸^2^MacNew Heart Disease Health-related Quality of Life Instrument.

Compared with the 336 persons, who did not respond to the survey or were excluded from the analysis, the 546 participants were significantly younger (68.5 ± 12.2 years versus 71.8 ± 13.4 years). No significant differences were found regarding gender and school education.

### 3.2 Associations between health literacy and sociodemographic variables

The subscale scores of the HLQ for the total sample and different subgroups are presented in **Table 2**. Compared with women, men showed a significantly better health literacy regarding ‘Navigating the healthcare system’. For the subscales ‘Actively managing my health’ and ‘Ability to find good health information’ a significant association with age was found: the younger the persons, the higher (better) the scores. Significantly positive associations between higher school education and better health literacy were found in most HLQ subscales except for the subscales ‘Feeling understood and supported by healthcare providers’and ‘Social support for health’. Persons with German nationality (92.9%) showed significantly poorer health literacy in the HLQ subscale ‘Social support for health’ compared with persons with non-German nationality (7.1%). Likewise, persons living alone had significantly lower scores in this subscale compared with individuals living with a partner.

**Table 2:** Scores of the Health Literacy Questionnaire (HLQ) subscales (means and standard deviations) stratified by gender, age, school education, nationality, and living alone.

	HLQ subscales								
	#1	#2	#3	#4	#5	#6	#7	#8	#9
**Total sample** (n=546)	3.14 (0.58)	2.90 (0.53)	2.85 (0.53)	3.10 (0.55)	2.55 (0.60)	3.66 (0.82)	3.64 (0.78)	3.42 (0.81)	3.54 (0.79)
Gender									
Male (n=396)	3.15 (0.57)	2.91 (0.51)	2.85 (0.53)	3.08 (0.55)	2.55 (0.61)	3.70 (0.81)	**3.69 (0.76)**	3.45 (0.81)	3.55 (0.77)
Female (n=150)	3.11 (0.62)	2.87 (0.59)	2.85 (0.55)	3.14 (0.57)	2.57 (0.56)	3.56 (0.84)	**3.50 (0.77)**	3.34 (0.81)	3.50 (0.83)
Age [years]									
< 60 (n=140)	3.21 (0.60)	2.96 (0.52)	**2.94 (0.55)**	3.06 (0.59)	2.60 (0.58)	3.82 (0.75)	3.70 (0.75)	**3.61 (0.75)**	3.67 (0.75)
60 - 69 (n=125)	3.05 (0.59)	2.84 (0.55)	**2.78 (0.50)**	3.04 (0.60)	2.54 (0.56)	3.65 (0.81)	3.66 (0.75)	**3.49 (0.77)**	3.56 (0.73)
70 - 79 (n=162)	3.15 (0.54)	2.89 (0.50)	**2.80 (0.50)**	3.11 (0.55)	2.56 (0.59)	3.59 (0.81)	3.59 (0.79)	**3.35 (0.81)**	3.50 (0.78)
≥ 80 (n=119)	3.12 (0.62)	2.90 (0.56)	**2.89 (0.58)**	3.19 (0.44)	2.51 (0.67)	3.59 (0.89)	3.60 (0.83)	**3.23 (0.87)**	3.41 (0.86)
Education									
< 9 years schooling (n=305)	3.10 (0.61)	**2.85 (0.58)**	**2.78 (0.57)**	3.12 (0.56)	**2.46 (0.61)**	**3.58 (0.86)**	**3.53 (0.81)**	**3.26 (0.84)**	**3.36 (0.81)**
≥ 9 years schooling (n=234)	3.19 (0.55)	**2.97 (0.46)**	**2.94 (0.48)**	3.07 (0.54)	**2.66 (0.57)**	**3.78 (0.76)**	**3.78 (0.71)**	**3.64 (0.73)**	**3.78 (0.69)**
Nationality									
German (n=507)	3.14 (0.78)	2.90 (0.53)	2.86 (0.53)	**3.08 (0.55)**	2.56 (0.60)	3.66 (0.81)	3.65 (0.77)	3.43 (0.80)	3.56 (0.76)
Non German (n=39)	3.06 (0.66)	2.89 (0.53)	2.70 (0.58)	**3.29 (0.50)**	2.46 (0.59)	3.61 (0.92)	3.49 (0.90)	3.30 (0.91)	3.32 (1.01)
Living alone									
Yes (n=121)	3.12 (0.63)	2.91 (0.54)	2.90 (0.57)	**2.97 (0.68)**	2.55 (0.62)	3.70 (0.81)	3.67 (0.80)	3.44 (0.83)	3.53 (0.82)
No (n=423)	3.14 (0.57)	2.90 (0.53)	2.84 (0.52)	**3.14 (0.50)**	2.55 (0.59)	3.65 (0.82)	3.63 (0.77)	3.42 (0.81)	3.54 (0.78)

Significant differences (T-test or analysis of variance, p<0.05) are highlighted in bold type.

### 3.3 Associations between health literacy and HRQOL

In the unadjusted regression analyses, significant associations between health literacy (independent variable) and HRQOL (dependent variable) were found for all HLQ and McNew questionnaire subscales (see **Table 3**, model 1). The highest regression estimates were found for the HLQ subscales ‘Having sufficient information to manage my health’ (ß = 0.63 – 0.71) and ‘Navigating the healthcare system’ (ß = 0.63 – 0.68). Lowest explained variance (adjusted R-square) was found for the HLQ subscale ‘Appraisal of health information’ (0.93% to 1.27%), while HLQ subscale ‘Navigating the healthcare system’ showed highest explained variance (17.46% to 22.74%).

**Table 3:** Linear regression models of health literacy (9 subscales of the Health Literacy Questionnaire) and health-related quality of life (3 subscales and total score of the MacNew Heart Disease Health-related Quality of Life Instrument).

	MacNew Heart Disease Health-related Quality of Life Instrument			
	Total	Physical	Emotional	Social
1 Feeling understood and supported by healthcare providers				
Model 1^1^	0.43 (0.28 – 0.58), <0.0001	0.39 (0.23 – 0.56), <0.0001	0.48 (0.32 – 0.63), <0.0001	0.42 (0.26 – 0.59), <0.0001
Model 2^2^	0.40 (0.25 – 0.55), <0.0001	0.35 (0.19 – 0.51), <0.0001	0.45 (0.30 – 0.61), <0.0001	0.39 (0.23 – 0.55), <0.0001
Model 3^3^	0.19 (0.10 – 0.28), <0.0001	0.12 (0.01 – 0.23), 0.0462	0.25 (0.16 – 0.34), <0.0001	0.17 (0.06 – 0.28), 0.0024
2 Having sufficient information to manage my health				
Model 1	0.67 (0.51 – 0.83), <0.0001	0.63 (0.46 – 0.81), <0.0001	0.71 (0.55 – 0.88), <0.0001	0.65 (0.48 – 0.82), <0.0001
Model 2	0.64 (0.48 – 0.79), <0.0001	0.60 (0.42 – 0.77), <0.0001	0.68 (0.52 – 0.85), <0.0001	0.61 (0.45 – 0.78), <0.0001
Model 3	0.24 (0.14 – 0.34), <0.0001	0.18 (0.05 – 0.31), 0.0062	0.28 (0.18 – 0.38), <0.0001	0.22 (0.10– 0.34), 0.0005
3 Actively managing my health				
Model 1	0.46 (0.29 – 0.62), <0.0001	0.46 (0.28 – 0.63), <0.0001	0.49 (0.32 – 0.66), <0.0001	0.46 (0.28 – 0.63), <0.0001
Model 2	0.40 (0.23 – 0.56), <0.0001	0.38 (0.20 – 0.56), <0.0001	0.44 (0.27 – 0.62), <0.0001	0.38 (0.21 – 0.56), <0.0001
Model 3	-0.01 (-0.11 – 0.10), 0.8686	-0.04 (-0.17 – 0.09), 0.5794	0.03 (-0.07 – 0.14), 0.5609	-0.02 (-0.15 – 0.11), 0.7458
4 Social support for health				
Model 1	0.53 (0.37 – 0.68), <0.0001	0.47 (0.29 – 0.64), <0.0001	0.62 (0.48 – 0.80), <0.0001	0.49 (0.36 – 0.65), <0.0001
Model 2	0.61 (0.45 – 0.76), <0.0001	0.56 (0.39 – 0.73), <0.0001	0.71 (0.55 – 0.87), <0.0001	0.59 (0.42 – 0.75), <0.0001
Model 3	0.18 (0.07 – 0.28), 0.0008	0.13 (-0.01 – 0.25), 0.0532	0.25 (0.15 – 0.35), <0.0001	0.16 (0.03 – 0.28), 0.0122
5 Appraisal of health information				
Model 1	0.21 (0.07 – 0.36), 0.0048	0.21 (0.05 – 0.38), 0.0100	0.23 (0.08 – 0.39), 0.0034	0.20 (0.04 – 0.36, 0.0139
Model 2	0.15 (0.01 – 0.30), 0.0408	0.14 (-0.02 – 0.30), 0.0920	0.19 (0.03 – 0.34), 0.0182	0.13 (-0.02 – 0.29), 0.0959
Model 3	-0.04 (-0.13 – 0.05), 0.3964	-0.07 (-0.18 – 0.04), 0.2138	0.00 (-0.09 – 0.09), 0.9891	-0.06 (-0.18 – 0.05), 0.2561
6 Ability to actively engage with healthcare providers				
Model 1	0.53 (0.43 – 0.63), <0.0001	0.50 (0.38 – 0.61), <0.0001	0.57 (0.47 – 0.67), <0.0001	0.52 (0.41 – 0.63), <0.0001
Model 2	0.51 (0.41 – 0.61), <0.0001	0.46 (0.35 – 0.57), <0.0001	0.56 (0.46 – 0.66), <0.0001	0.49 (0.38 – 0.60), <0.0001
Model 3	0.19 (0.12 – 0.26), <0.0001	0.14 (0.05 – 0.22), 0.0015	0.23 (0.16 – 0.30), <0.0001	0.18 (0.10 – 0.26), <0.0001
7 Navigating the healthcare system				
Model 1	0.65 (0.55 – 0.75), <0.0001	0.63 (0.51 – 0.74), <0.0001	0.68 (0.58 – 0.79), <0.0001	0.64 (0.53 – 0.75), <0.0001
Model 2	0.62 (0.51 – 0.72), <0.0001	0.59 (0.48 – 0.70), <0.0001	0.66 (0.55 – 0.77), <0.0001	0.60 (0.49 – 0.71), <0.0001
Model 3	0.23 (0.16 – 0.30), <0.0001	0.20 (0.11 – 0.30), <0.0001	0.25 (0.18 – 0.33), <0.0001	0.23 (0.14 – 0.32), <0.0001
8 Ability to find good health information				
Model 1	0.47 (0.36 – 0.57), <0.0001	0.46 (0.35 – 0.58), <0.0001	0.49 (0.38 – 0.59), <0.0001	0.47 (0.36 – 0.58), <0.0001
Model 2	0.43 (0.32 – 0.54), <0.0001	0.40 (0.29 – 0.52), <0.0001	0.47 (0.36 – 0.58), <0.0001	0.42 (0.30 – 0.53), <0.0001
Model 3	0.12 (0.05 – 0.19), 0.0006	0.09 (0.01 – 0.18), 0.0367	0.15 (0.08 – 0.22), 0.0001	0.11 (0.03 – 0.20), 0.0090
9 Understanding health information well enough to know what to do				
Model 1	0.48 (0.37 – 0.59), <0.0001	0.48 (0.36 – 0.60), <0.0001	0.50 (0.39 – 0.61), <0.0001	0.49 (0.37 – 0.60), <0.0001
Model 2	0.43 (0.32 – 0.54), <0.0001	0.40 (0.28 – 0.53), <0.0001	0.47 (0.35 – 0.59), <0.0001	0.43 (0.31 – 0.55), <0.0001
Model 3	0.08 (0.01 – 0.16), 0.0254	0.06 (-0.03 – 0.15), 0.2022	0.10 (0.03 – 0.18), 0.0071	0.09 (-0.01 – 0.18), 0.0534

^1^Model 1: Unadjusted; ^2^Model 2: Adjusted for age, gender, school education, nationality, living alone; ^3^Model 3: Model 2 plus adjustment for depression (continuous PHQ-9 score)

ß-coefficients, 95% confidence interval and p-value are displayed for each model.

After adjusting the models for sociodemographic covariables, the beta coefficients slightly decreased, but in most of the models the association between health literacy and HRQOL remained significant, except the association between ‘Appraisal of health information’ and the physical and social subscale of the McNew questionnaire (see **Table 3**, model 2). Similar with the unadjusted regression models, lowest explained variance was found for the HLQ subscale ‘Appraisal of health information’ (5.46% to 7.42%), and highest for HLQ subscale ‘Navigating the healthcare system’ (22.38% to 25.32%).

The inclusion of the continuous PHQ score in the models resulted in a considerable decrease of the beta coefficients in all models (see **Table 3**, model 3). Several associations with the HLQ lost statistical significance, specifically the subscales ‘Actively managing my health’, ‘Appraisal of health information’ and ‘Understanding health information well enough to know what to do’. The explained variance of the models ranged between 56.14% and 72.15%.

### 3.4 Mediation analyses

Since the results of the fully adjusted regression models indicated a partial or even complete mediation by depression, further mediation analyses were conducted according to the model shown in **Figure 1**.

The linear regression between HLQ (independent variable) and PHQ (dependent variable) (path a) resulted in significant inverse associations with beta coefficients ranging between -1.18 and -2.51, depending on the HLQ subscale (data not shown). Similarly, the linear regression between PHQ (independent variable) and McNew scores (dependent variable) (path b) revealed significant results with beta coefficients of -0.18 (Subscales ‘Physical’, ‘Social’ and ‘Total’) and -0.20 (Subscale ‘Emotional’). Path c’ refers to the direct association between health literacy and HRQOL estimated by the fully adjusted regression models (see **Table 3**, Models 3).

**Figure 2:**
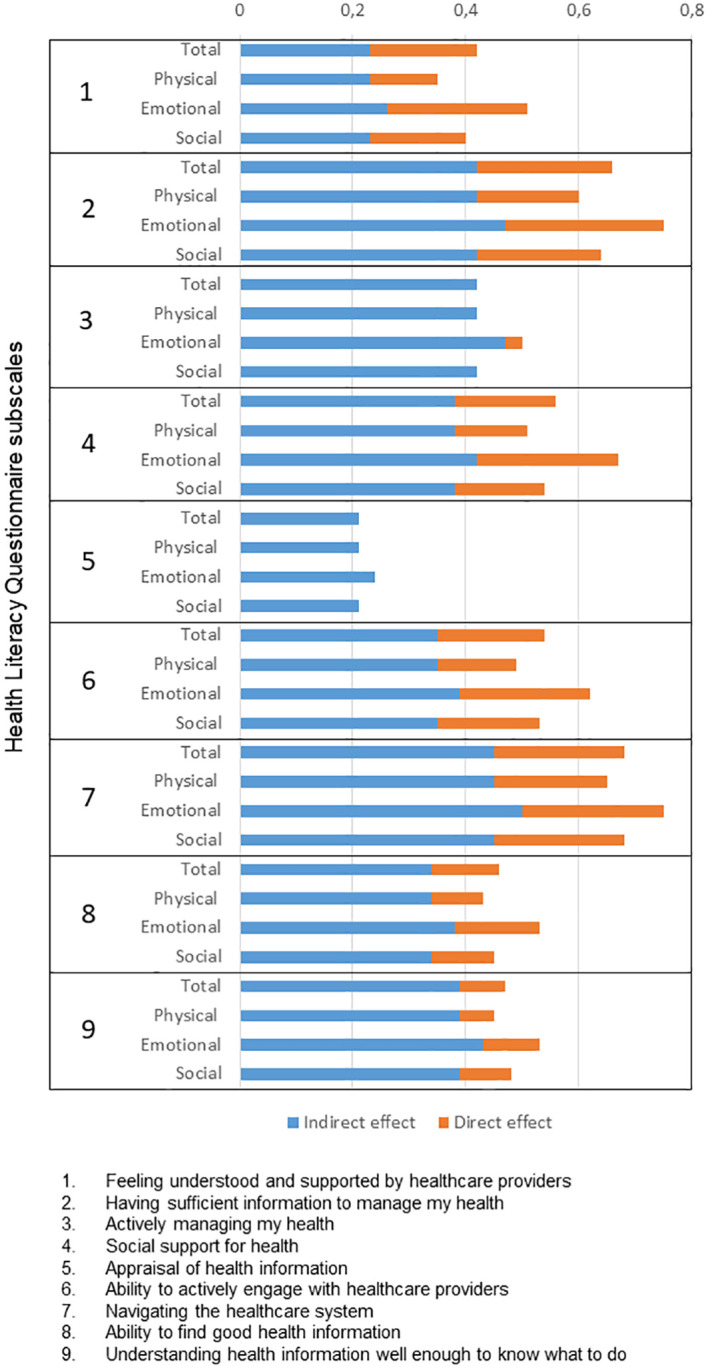
Associations between the Health Literacy Questionnaire (9 subscales) and the MacNew Heart Disease Quality of Life Instrument (total score and 3 subscales). Each total effect is shown as its respective proportion of direct and indirect effect (the related estimates are shown in **Supplementary Table 1**).

The total effects of health literacy ranged between 0.14 (McNew subscale “Physical” and HLQ subscale ‘Appraisal of health information’) and 0.75 (McNew subscale “Emotional” and HLQ subscales ‘Having sufficient information to manage my health’ and ‘Navigating the healthcare system’) (see **Figure 2**; **Supplementary Table 1**). The indirect effect of depression in the models (product of the beta estimates of path a and b) ranged between 0.21 and 0.50 and accounted for more than 50% of the total effect in all models (see **Figure 2**; **Supplementary Table 1**). A complete mediation effect of depression was found for the HLQ subscales ‘Actively managing my health’ and ‘Appraisal of health information’.

## 4 Discussion

In the present survey of 546 patients with acute myocardial infarction two to 18 months after the acute event, most dimensions of health literacy were negatively associated with poor education. Regression models revealed significant associations between poor health literacy and poor HRQOL in all subscales, which mostly were independent of sociodemographic variables. However, more than 50% of the association between health literacy and HRQOL was mediated by depression. A complete mediating effect was found for the HLQ subscales ‘Actively managing my health’ and ‘Appraisal of health information’.

In the present analyses poor school education turned out to be the most relevant sociodemographic variable associated with poor health literacy in patients with acute myocardial infarction. This finding is in line with results from studies on patients with coronary artery disease (4). However, this study provides a more differentiated depiction of the association of other sociodemographic variables with health literacy. Contrary to the results from other studies, men did not generally show lower levels of health literacy (4), but even had better scores in the subscale ‘Navigating the healthcare system’ than women. Older persons showed limitations regarding an active health management and finding good health information. Moreover, persons with German nationality or persons living alone had poorer social support for health, compared with persons with non-German nationality and individuals living with a partner, respectively. These results provide valuable information for tailoring interventions aimed at improving health literacy to the needs of specific subgroups.

Overall, the association between health literacy and HRQOL found in other studies was supported by the findings of the present study (3, 4). More precisely, a lack of sufficient information to manage ones health and problems navigating the healthcare system were the dimensions of health literacy with the strongest and direct effects on HRQOL. A significant independent association (without adjustment for depression) between the HLQ subscale ‘Navigating the healthcare system’ and HRQOL was also found in a study on 150 people in cardiac rehabilitation (18).

The present study showed that the association between health literacy and HRQOL was considerably mediated by depression. Mediating effects of self-efficacy or self-care activities have been reported in patients with coronary heart disease (34) and diabetes (35). Also, depression was shown to mediate the association between health literacy and medication adherence and HRQOL (9), diet adherence (11) and self-care (10) in studies with different health conditions. The mediating effect of depression in the present study is an important finding, since about 30% of patients with acute myocardial infarction suffer from major depression or depressive symptoms (36). Adverse effects of depression on the HRQOL of patients with acute myocardial infarction may be reduced by early diagnosis and treatment. However, these patients often remain undiagnosed and untreated (37, 38). The results from the present study also indicate that improving health literacy may reduce depressive symptoms and subsequently improve HRQOL. However, longitudinal studies and randomized controlled trials are required to investigate causal relationships between health literary, HRQOL and depression in the future.

To our knowledge, the present study is the first which provides a comprehensive investigation of the associations between health literacy, disease-specific HRQOL, and depression in patients with acute myocardial infarction. Strengths of this study are the large number of well-characterized consecutive acute myocardial infarction-patients from a population-based registry studied prospectively, and the assessment of disease-specific HRQOL and multi-dimensional health literacy. Limitations of the study include that no clinical diagnosis of depression was available. The finding that the included participants were significantly younger than the non-responding persons indicates a selection bias. Futhermore, individuals with poor health literacy, poor HRQOL or higher levels of depression may have been less likely to participate in the follow-up survey. The study participants had their acute myocardial infarction during the SARS-CoV-2 pandemic and it is possible that this situation may have adversely affected study outcomes such as depression and HRQOL. Furthermore, due to the cross-sectional study design, causal relationships between health literacy, HRQOL and depression cannot be deduced. Moreover, the number of significant results may have been affected by multiple testing. Thus, future studies with longitudinal design and an adjustment for multiple testing are strongly recommended. Finally, the results may not be generalizable to other age-groups and all ethnic groups.

In conclusion, the present study found a significant association between health literacy and HRQOL in patients with acute myocardial infarction which was partially or completely mediated by depression. The different dimensions of health literacy may be considered for interventions aimed at improving HRQOL, which should be tailored to subgroups of patients such as elderly and poorly educated and evaluated in randomized controlled studies. Considering both, health literacy and depression, may improve the HRQOL of patients with acute myocardial infarction in the future.

## Data availability statement

The datasets presented in this article are not readily available because of ethical and privacy restrictions. Requests to access the datasets should be directed to: CM, christine.meisinger@med.uni-augsburg.de.

## Ethics statement

The studies involving humans were approved by Ethics committee of the Bavarian Medical Association (Bayerische Landesärztekammer). The studies were conducted in accordance with the local legislation and institutional requirements. The participants provided their written informed consent to participate in this study.

## Author contributions

IK: Formal analysis, Writing – original draft. SF: Formal analysis, Supervision, Writing – review & editing. PR: Resources, Writing – review & editing. JL: Resources, Writing – review & editing. CM: Conceptualization, Methodology, Writing – review & editing. TS: Conceptualization, Investigation, Writing – review & editing.
