# Supplementary material for: Depression mediates the association between health literacy and health-related quality of life after myocardial infarction
Source: Front Psychiatry. 2024 Feb 14;15:1341392. doi: 10.3389/fpsyt.2024.1341392 (PMC10899501; doi:10.3389/fpsyt.2024.1341392)
Supplement: Supplementary file 1 [file Table_1.docx]

Table S1: Results of the Mediation analyses

|  | MacNew Heart Disease Health-related Quality of Life Instrument | | | |
| --- | --- | --- | --- | --- |
|  | Total | Physical | Emotional | Social |
| 1 Feeling understood and supported by healthcare providers | | | | |
| Indirect effect: a*b | 0.23 | 0.23 | 0.26 | 0.23 |
| Direct effect: c’ | 0.19 | 0.12 | 0.25 | 0.17 |
| Total effect: c’ + (a*b) | 0.42 | 0.35 | 0.51 | 0.40 |
| Indirect/total | 54.76% | 65.71% | 50.98% | 57.50% |
| 2 Having sufficient information to manage my health | | | | |
| Indirect effect: a*b | 0.42 | 0.42 | 0.47 | 0.42 |
| Direct effect: c’ | 0.24 | 0.18 | 0.28 | 0.22 |
| Total effect: c’ + (a*b) | 0.66 | 0.60 | 0.75 | 0.64 |
| Indirect/total | 63.64% | 70.00% | 62.67% | 65.63% |
| 3 Actively managing my health | | | | |
| Indirect effect: a*b | 0.42 | 0.42 | 0.47 | 0.42 |
| Direct effect: c’ | -0.01 | -0.04 | 0.03 | -0.02 |
| Total effect: c’ + (a*b) | 0.41 | 0.38 | 0.50 | 0.40 |
| Indirect/total | 100.02% | 110.53% | 94.00% | 105.00% |
| 4 Social support for health | | | | |
| Indirect effect: a*b | 0.38 | 0.38 | 0.42 | 0.38 |
| Direct effect: c’ | 0.18 | 0.13 | 0.25 | 0.16 |
| Total effect: c’ + (a*b) | 0.56 | 0.51 | 0.67 | 0.54 |
| Indirect/total | 67.86% | 74.51% | 62.69% | 70.37% |
| 5 Appraisal of health information | | | | |
| Indirect effect: a*b | 0.21 | 0.21 | 0.24 | 0.21 |
| Direct effect: c’ | -0.04 | -0.07 | 0.00 | -0.06 |
| Total effect: c’ + (a*b) | 0.17 | 0.14 | 0.24 | 0.15 |
| Indirect/total | 123.53% | 150.00% | 100.00% | 140.00% |
| 6 Ability to actively engage with healthcare providers | | | | |
| Indirect effect: a*b | 0.35 | 0.35 | 0.39 | 0.35 |
| Direct effect: c’ | 0.19 | 0.14 | 0.23 | 0.18 |
| Total effect: c’ + (a*b) | 0.54 | 0.49 | 0.62 | 0.53 |
| Indirect/total | 64.81% | 71.43% | 62.90% | 66.04% |
| 7 Navigating the healthcare system | | | | |
| Indirect effect: a*b | 0.45 | 0.45 | 0.50 | 0.45 |
| Direct effect: c’ | 0.23 | 0.20 | 0.25 | 0.23 |
| Total effect: c’ + (a*b) | 0.68 | 0.65 | 0.75 | 0.68 |
| Indirect/total | 66.18% | 69.23% | 66.67% | 66.18% |
| 8 Ability to find good health information | | | | |
| Indirect effect: a*b | 0.34 | 0.34 | 0.38 | 0.34 |
| Direct effect: c’ | 0.12 | 0.09 | 0.15 | 0.11 |
| Total effect: c’ + (a*b) | 0.46 | 0.43 | 0.43 | 0.45 |
| Indirect/total | 73.91% | 79.07% | 88.37% | 75.56% |
| 9 Understanding health information well enough to know what to do | | | | |
| Indirect effect: a*b | 0.39 | 0.39 | 0.43 | 0.39 |
| Direct effect: c’ | 0.08 | 0.06 | 0.10 | 0.09 |
| Total effect: c’ + (a*b) | 0.47 | 0.45 | 0.53 | 0.48 |
| Indirect/total | 82.98% | 86.67% | 81.13% | 81.25% |
